# Supplementary material for: Consensus-based qubit configuration optimization for variational algorithms on neutral atom quantum systems
Source: npj Quantum Inf. 2025 Nov 27;11(1):186. doi: 10.1038/s41534-025-01132-8 (PMC12660136; doi:10.1038/s41534-025-01132-8)
Supplement: Supplementary file 1 — Supplementary Information [file 41534_2025_1132_MOESM1_ESM.pdf]

SUPPLEMENTARY INFORMATION

Gradient-free optimization algorithm comparison

This work primarily suggests a gradient-free method for configuration optimization. The CBO is selected as it facilitates a high degree of parallelization and is straightforward to implement on the neutral atom system. Nevertheless, it is useful to compare the performance of the CBO to other gradient-free optimization techniques. In this appendix, we compare to a basic genetic algorithm (implemented using the PyGAD Python library [1]) and a Bayesian optimization algorithm (implemented using the BayesianOptimization Python library [2]). The hyperparameters of these algorithms are similar to the standard examples, as provided by the libraries. These algorithms offer a fair comparison, as they both sample points in the configuration space (like the agents in the CBO) and construct new configurations based on their results.

We ensure that each algorithm starts with the same target Hamiltonian H_{targ}, same initial configurations, $N_{\mathrm{in}} = 30$, and halts after a fixed number of function evaluations ($= N_{\mathrm{out}} \times K = 30 \times 12$). This halting criterium is chosen as it roughly relates to the total time spent on the quantum system. For the final configurations, the pulses are optimized with $N_{\mathrm{final}} = 100$. This experiment is repeated for 50 target Hamiltonians, leading to a distribution on the found energy errors.

The results of this simulation are shown in Supplementary Figure 1. These results indicate that the CBO generally outperforms both the Bayesian and genetic algorithms. The Bayesian algorithm is able to find low-error configurations, likely because it also samples in the simplex of the initial configurations. Outside some outliers, the mutating nature of the genetic algorithm does not seem to be a good fit for the configuration optimization problem (even after heuristically adjusting the hyperparameters). This simple but illustrative analysis supports the choice for the CBO as a gradient-free optimization algorithm.

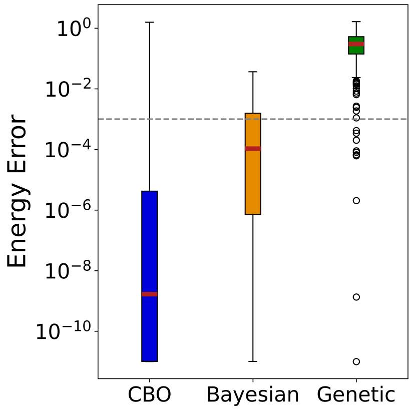

Supplementary Figure 1. Comparison of gradient-free optimization algorithms for configuration optimization. 50 problems with same target Hamiltonians, initial positions, and number of function evaluations ($= N_{\mathrm{out}} \times K$) for each algorithm. $N_{\mathrm{in}} = 30$ and $N_{\mathrm{final}} = 100$. The consensus-based optimization (CBO) algorithm outperforms both the Bayesian and genetic algorithm.

REFERENCES

[1] A. F. Gad, Multimedia Tools and Applications , 1 (2023).
[2] F. Nogueira, "Bayesian Optimization: Open source constrained global optimization tool for Python," (2014–).
